# Short-term exposure to ambient air pollution and pneumonia hospital admission among patients with COPD: a time-stratified case-crossover study

**DOI:** 10.1186/s12931-022-01989-9

**Published:** 2022-03-26

**Authors:** Wenfeng Lu, Qi Tian, Ruijun Xu, Chenghui Zhong, Lan Qiu, Han Zhang, Chunxiang Shi, Yuewei Liu, Yun Zhou

**Affiliations:** 1 State Key Laboratory of Respiratory Disease, The First Affiliated Hospital of Guangzhou Medical University, Guangzhou, 510120 Guangdong China; 2 School of Public Health, Guangzhou Medical University, Guangzhou, 511436 Guangdong China; 3 Guangzhou Health Technology Identification and Human Resources Assessment Center, Guangzhou, 510080 Guangdong China; 4 Department of Epidemiology, School of Public Health, Sun Yat-Sen University, Guangzhou, 510080 Guangdong China; 5 National Meteorological Information Center, China Meteorological Administration, Beijing, 100081 China

**Keywords:** Pneumonia, COPD patients, Air pollution, Hospital admission

## Abstract

**Background:**

Pneumonia is a major contributor to hospital admission for patients with chronic obstructive pulmonary disease (COPD). However, evidence for acute effects of ambient air pollution exposure on pneumonia hospital admission among patients with COPD is scarce. We aimed to examine the association between short-term exposure to ambient air pollution and pneumonia hospital admission among patients with COPD.

**Methods:**

We enrolled COPD cases aged ≥ 60 years old and further filtered those who were admitted into hospitals from pneumonia during 2016–2019 in Guangdong province, China for main analysis. A time-stratified case-crossover design was applied to investigate the association and conditional logistic regression model was used for data analysis. We performed inverse distance weighting method to estimate daily individual-level exposure on particulate matter with an aerodynamic diameter ≤ 2.5 μm (PM_2.5_), particulate matter with an aerodynamic diameter ≤ 10 μm (PM_10_), sulfur dioxide (SO_2_), nitrogen dioxide (NO_2_), carbon monoxide (CO), and ozone (O_3_) based on personal residential addresses.

**Results:**

We included 6473 pneumonia hospital admissions during the study period. Each interquartile range (IQR) increase in PM_2.5_ (lag 2; IQR, 22.1 μg/m^3^), SO_2_ (lag 03; IQR, 4.2 μg/m^3^), NO_2_ (lag 03; IQR, 21.4 μg/m^3^), and O_3_ (lag 04; IQR, 57.9 μg/m^3^) was associated with an odds ratio in pneumonia hospital admission of 1.043 (95% CI: 1.004–1.083), 1.081 (95% CI: 1.026–1.140), 1.045 (95% CI: 1.005–1.088), and 1.080 (95% CI: 1.018–1.147), respectively. Non-linear trends for PM_2.5_, PM_10_, and SO_2_ were observed in the study. Sex, age at hospital admission, and season at hospital admission did not modify the associations.

**Conclusions:**

We found significantly positive associations of short-term exposure to PM_2.5_, SO_2_, NO_2_, and O_3_ with pneumonia hospital admission among COPD patients. It provides new insight for comprehensive pneumonia prevention and treatment among COPD patients.

**Supplementary Information:**

The online version contains supplementary material available at 10.1186/s12931-022-01989-9.

## Introduction

Chronic obstructive pulmonary disease (COPD) is a group of lung diseases that causes breathing-related problems with dyspnea and cough, which has become the third leading cause of death worldwide since 2017 [1, 2]. COPD is characterized by irreversible damage in the lungs that affects the exchange of oxygen and carbon dioxide, and can easily lead to complications. People suffer from COPD frequently accompanied by various complications such as lower respiratory infection, lung cancer, psychiatric diseases, and cardiovascular diseases [3, 4]. In the recommendation of Global Initiative for Chronic Obstructive Lung Disease, management and treatment for COPD requires evaluating the presence of complications in addition to COPD itself [5].

Pneumonia is one of the major and common complications of COPD, caused by infectious agents including viruses, bacteria, fungi, or other pathogenic microorganisms [6]. On the basis of epidemiological and experimental studies, COPD weakens patients’ respiratory systems, and makes it easier for pneumonia to swoop in [7, 8]. It was reported that newly diagnosed COPD patients have 15 times higher risk for pneumonia compared to those without COPD [9] and worse clinical presentation along with more intensive care was needed among patients with COPD [10, 11]. As pneumonia among COPD patients can cause an increased risk of respiratory failure and become life threatening beyond just shortness of breath and limitations of activities, it is necessary to control the pneumonia among COPD patients.

In addition to the infectious agents, pneumonia can also indirectly caused by environmental factors induced weakened immune systems [12]. Air pollution is one of the most important environmental factors that increases the susceptibility to pathogens via impairing epithelial cell functions and decreasing mucociliary clearance. Previous studies have found that exposure to ambient air pollutants can increase the risk of pneumonia [13, 14]. However, there was limited studies addressing the relation among patients with COPD. Only one case–control study with hospitalized cases in Taiwan explored that COPD patients had a higher risk of pneumonia occurrence by air pollution, which was performed with a small sample size and carried out the exposure assessment by fixed monitoring stations [15].

Therefore, we conducted a large time-stratified case-crossover study from 2016 to 2019 in Guangdong province, China, to estimate the acute adverse effect of ambient air pollution on hospital admission for pneumonia among patients with COPD. We hypothesized that short-term exposure to certain ambient air pollutants including particulate matter with an aerodynamic diameter ≤ 2.5 μm (PM_2.5_), particulate matter with an aerodynamic diameter ≤ 10 μm (PM_10_), sulfur dioxide (SO_2_), nitrogen dioxide (NO_2_), carbon monoxide (CO), and ozone (O_3_), would be associated with increased risk of hospital admission for pneumonia among patients with COPD.

## Methods

### Study population and outcome

We firstly identified a total of 64,815 COPD patients aged 60 years or older who lived in Guangdong province, China during January 1, 2016 to December 31, 2019 based on the codes of International Statistical Classification of Diseases and Related Health Problems 10th Revision (ICD-10) in their electronic medical records (ICD-10 codes: J40-J44, J47). The medical data on hospital admission was derived from the National Health Statistics Network Direct Reporting System of the Guangzhou Health Technology Identification and Human Resources Assessment Center. The online system includes all the medical institutions with in-patient service in Guangzhou, China and requires them to report daily medical data. In addition, all the records under good quality control are subsequently saved into the system. Second, we further retrieved those patients with their records on admission from pneumonia (ICD-10 codes: J12-J18) and obtained 6743 pneumonia cases among patients with COPD. Information on sex, age at hospital admission, residential address, and date of hospital admission was extracted from electronic medical records.

### Study design

A time-stratified case-crossover design was applied for assessing the association between short-term exposure to air pollution and pneumonia hospital admission among patients with COPD, which is one of the most widely used designs for the acute health effect evaluation of air pollution [16]. In this design, case day was defined as the date of hospital admission and each subject serves as his or her own control in the same stratum (e.g., 1 month). The control days were defined as the other same days of the week within the same month and year of case day, which generated 3 or 4 days for specified referent window. This design was proposed to control for potential confounding factors including individual covariates, day of the week, seasonality, and long-term trend [17, 18]. For example, if a pneumonia case was admitted into the hospital on September 12, 2016 (Monday), the case day was defined as September 12, 2016, while the control days were defined as the all the other Mondays in September (i.e., September 5, September 19, and September 26, 2016).

### Exposure assessment

Daily data on 24-h average concentrations of PM_2.5_, PM_10_, SO_2_, NO_2_, CO and daily maximum 8-h average concentration of O_3_ in Guangdong province were measured by state-controlled air quality monitoring stations and were obtained from the National Urban Air Quality Real-Time Publishing Platform of the China Meteorological administration in China during 2016–2019. We collected the daily air pollution data based on the 105 monitoring stations located in Guangdong province and 9 monitoring stations in neighboring provinces.

The inverse distance weighting (IDW) method was performed to estimate the daily individual-level exposure to ambient air pollution, which is an effective method for air pollution exposure estimation based on spatial distribution [19, 20]. The daily air pollution data from monitoring stations was assigned for each case days and control days based on residential addresses. We geocoded the locations of monitoring stations and residential addresses of pneumonia cases by Baidu Map API [21]. For each subject, we set up a proper buffer distance (25 km), where a case’s residential address was taken as the center and calculated the inverse squared distance (1/*d*^*2*^) weighted concentrations of the center at all monitoring stations within the buffer. In order to assess the lag effect of air pollution, the singled day lagged air pollution exposures were selected from 0 to 4 days and the moving average day lagged exposures (lag 01 to lag 04) were also assessed. For example, lag 0 exposure stands for the daily exposures to air pollution on the day of hospital admission, while lag 01 exposure stands for the mean of daily exposure on the day of hospital admission and its previous day. We excluded those pneumonia cases (N = 514) without a monitor station around 25 km near their residential addresses.

### Covariates

Gridded data (spatial resolution: 0.0625° × 0.0625°, temporal resolution: 1 day) on meteorological conditions in Guangdong province, China during 2016–2019 were acquired from the National Meteorological Information Center. Given the potential confounding effects of meteorological conditions, we assigned temperature and relative humidity to individuals for their case days and control days based on the geocoded residential addresses, and included them in all the models. The lag effects for meteorological conditions were also considered in the analysis as air pollution exposure. Apart from the meteorological condition, the frequency of hospital admission was also included in all the models to control for error of repeated measurement. Owing to the characteristic of case-crossover design (designed for self-control) [16], we did not consider individual covariates as confounding factors, for they did not vary substantially within a stratum.

### Statistical analysis

We used Spearman’s correlation coefficients to assess the strength and direction of the linear relationships between air pollutants and meteorological parameters. The strength of correlation was described by different descriptors based on the absolute magnitude for the observed Spearman correlation coefficients (weak: 0.00–0.39; moderate: 0.40–0.69; strong: 0.70–1.00) [22, 23]. The association between short-term exposure to air pollutants with different lag periods and pneumonia hospital admission among COPD patients was investigated using a conditional logistic regression model by pairing the exposures on case days and control days. All the models were adjusted for daily meteorological conditions with a smoothing function of natural cubic spline (temperature and relative humidity with degrees of freedom [*df*] of 3 for each) and the frequency of hospital admission to control for potential confounding factors. The results were reported as the estimated odds ratios (OR) for pneumonia hospital admission and their 95% confidence intervals (CI) with per interquartile range (IQR) increased. Exposure–response associations with natural cubic spline function were used to illustrate the nonlinear relation for air pollution exposure (*df* of 3) on pneumonia hospital admission among patients with COPD.

We also conducted stratified analyses to assess potential effect modification by sex (male, female), age at hospital admission (< 85 years, ≥ 85 years), and season at hospital admission (warm: May to October; cool: November to December, January to April) on the associations between air pollution exposure and pneumonia among patients with COPD. We applied a separate conditional logistic regression model for each subgroup and examined the differences within each group using the two-sample test [24]:

$$z=\frac{{\beta }_{1}-{\beta }_{2}}{\sqrt{{{SE}_{2}}^{2}+{{SE}_{2}}^{2}}}$$

where *β* = point estimates of association for air pollution exposure on pneumonia among patients with COPD derived from two categories; *SE* = their respective standard errors.

To test the robustness of our main results, we performed sensitivity analyses with two-pollutant models that included other pollutants one by one. Likelihood ratio test was used to compare the difference between the single- and two-pollutant models. All analyses were performed using the R software (version 4.0.5) [25]. All *p* values were two-sided, and a *p* value < 0.05 was considered statistically significant.

## Results

During 2016 to 2019, a total of 6473 patients with COPD who were hospitalized for pneumonia were included in this analysis. Approximate 9.5% had more than two admission records, resulting in 9431 case days and 31,914 control days (Table 1). Of the 9431 records, 68.3% were male and the mean age at hospital admission was 79.1 years. 55.0% of the cases were admitted in the cool season and 34.6% were due to the bacterial pneumonia.

**Table 1:** Baseline characteristics of study population in Guangdong province, China during 2016–2019

Baseline Characteristic	Values
Pneumonia hospital admissions, n (ICD-10 code: J12-J18)	6473
Case days, n	9431
Control days, n	31,914
Sex, n (%)	
Male	6438 (68.3)
Female	2993 (31.7)
Age at hospital admission	
Mean (SD)	79.1 (8.6)
Median (IQR)	80.0 (12.0)
n (%)	
< 85 year	6682 (70.9)
≥ 85 year	2749 (29.1)
Season at hospital admission, n (%) ^a^	
Warm	4246 (45.0)
Cool	5185 (55.0)
No. of hospital admission, n	
1	4836 (74.7)
2	1021 (15.8)
≥ 3	616 (9.5)
Main type of pneumonia, n (%)	
Bacterial pneumonia (ICD-10 code: J15)	3260 (34.6)
Pneumonia, organism unspecified (ICD-10 code: J18)	6152 (65.4)

*ICD* International Classification of Diseases, *SD* standard deviation, *IQR* interquartile range

^a^Warm season: from May to October; Cool season: November to December, January to April

The distributions of ambient air pollutants and meteorological condition during 9431 case days and 31,914 control days are shown in Table 2. The daily average concentrations for PM_2.5_, PM_10_, SO_2_, NO_2_, CO and daily maximum 8-h average concentration for O_3_ were 32.1 μg/m^3^, 53.8 μg/m^3^, 8.2 μg/m^3^, 46.5 μg/m^3^, 0.85 μg/m^3^, and 94.9 μg/m^3^, respectively. Moderate to strong positive correlations were observed between each two pollutants for PM_2.5_, PM_10_, NO_2_, SO_2_ and CO, except that weak correlation between SO_2_ and CO. O_3_ was negatively associated with CO and positively associated with other pollutants in weak correlations. Temperature was positively associated with O_3_, and negatively associated with other air pollutants, while all air pollutants were negatively correlated with relative humidity (Table 3).

**Table 2:** Distribution of air pollutants and meteorological conditions on case days in Guangdong, China during 2016–2019

Variable	Mean	SD	P_5_	P_25_	Median	P_75_	P_95_	IQR
Air pollutant								
PM_2.5_, μg/m^3^	32.1	17.9	11.5	19.2	28.0	40.9	65.2	21.7
PM_10_, μg/m^3^	53.8	27.3	21.5	34.1	46.9	68.0	108.1	33.9
SO_2_, μg/m^3^	8.2	3.7	4.1	5.4	7.3	9.9	15.2	4.5
NO_2_, μg/m^3^	46.5	21.0	19.4	32.7	42.4	55.9	86.4	23.2
CO, mg/m^3^	0.85	0.23	0.55	0.70	0.80	0.95	1.27	0.26
O_3_, μg/m^3^	94.9	50.5	20.7	58.4	89.0	125.8	190.3	67.4
Meteorological condition								
Temperature, °C	23.8	5.4	14.4	19.8	24.3	28.3	31.2	8.5
Relative humidity, %	76.6	13.2	50.1	69.4	80.3	86.7	91.8	17.2

*SD* standardized deviation, *P*_*5*_ the 5th percentile, *P*_*25*_ the 25th percentile, *P*_*75*_ the 75th percentile, *P*_*95*_ the 95th percentile, *IQR* interquartile range, *PM*_*2.5*_ particulate matter with an aerodynamic diameter ≤ 2.5 µm, *PM*_*10*_ particulate matter with an aerodynamic diameter ≤ 10 µm, *SO*_*2*_ sulfur dioxide, *NO*_*2*_ nitrogen dioxide, *CO* carbon monoxide, *O*_*3*_ ozone

**Table 3:** Spearman’s correlation coefficients between air pollutants and meteorological conditions on case days^a^

	PM_2.5_	PM_10_	SO_2_	NO_2_	CO	O_3_	Temperature
PM_10_	0.96	–	–	–	–	–	–
SO_2_	0.60	0.63	–	–	–	–	–
NO_2_	0.67	0.69	0.43	–	-	-	-
CO	0.55	0.50	0.26	0.60	–	–	–
O_3_	0.34	0.39	0.36	0.07	− 0.15	–	–
Temperature	− 0.28	− 0.21	− 0.03	− 0.27	− 0.43	0.46	–
Relative humidity	− 0.50	− 0.52	− 0.53	− 0.16	− 0.15	− 0.43	0.26

*PM*_*2.5*_ particulate matter with an aerodynamic diameter ≤ 2.5 µm, *PM*_*10*_ particulate matter with an aerodynamic diameter ≤ 10 µm, *SO*_*2*_ sulfur dioxide, *NO*_*2*_ nitrogen dioxide, *CO* carbon monoxide, *O*_*3*_ ozone

^a^All pairwise correlation coefficients were statistically significant (*p* < 0.001)

**Fig. 1:**
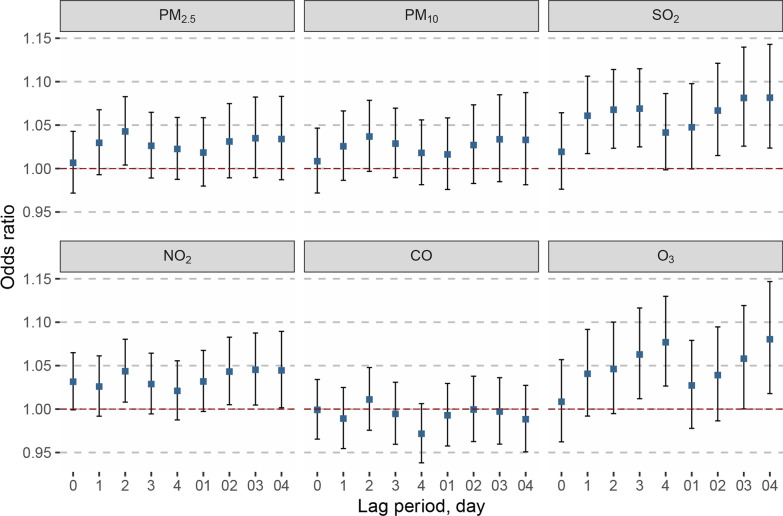
Adjusted ORs (95% CIs) for pneumonia hospital admission among COPD patients with air pollutants exposure. ORs for PM_2.5_, PM_10_, SO_2_, NO_2_, CO, and O_3_ at different lag periods were estimated using conditional logistic regression models, adjusting for temperature, relative humidity and number of admission records. *OR* odds ratio, *CI* confidence interval, *COPD* chronic obstructive pulmonary disease, *PM*_*2.5*_ particulate matter with an aerodynamic diameter ≤ 2.5 µm, *PM*_*10*_ particulate matter with an aerodynamic diameter ≤ 10 µm, *SO*_*2*_ sulfur dioxide, *NO*_*2*_ nitrogen dioxide, *CO* carbon monoxide, *O*_*3*_ ozone

**Fig. 2:**
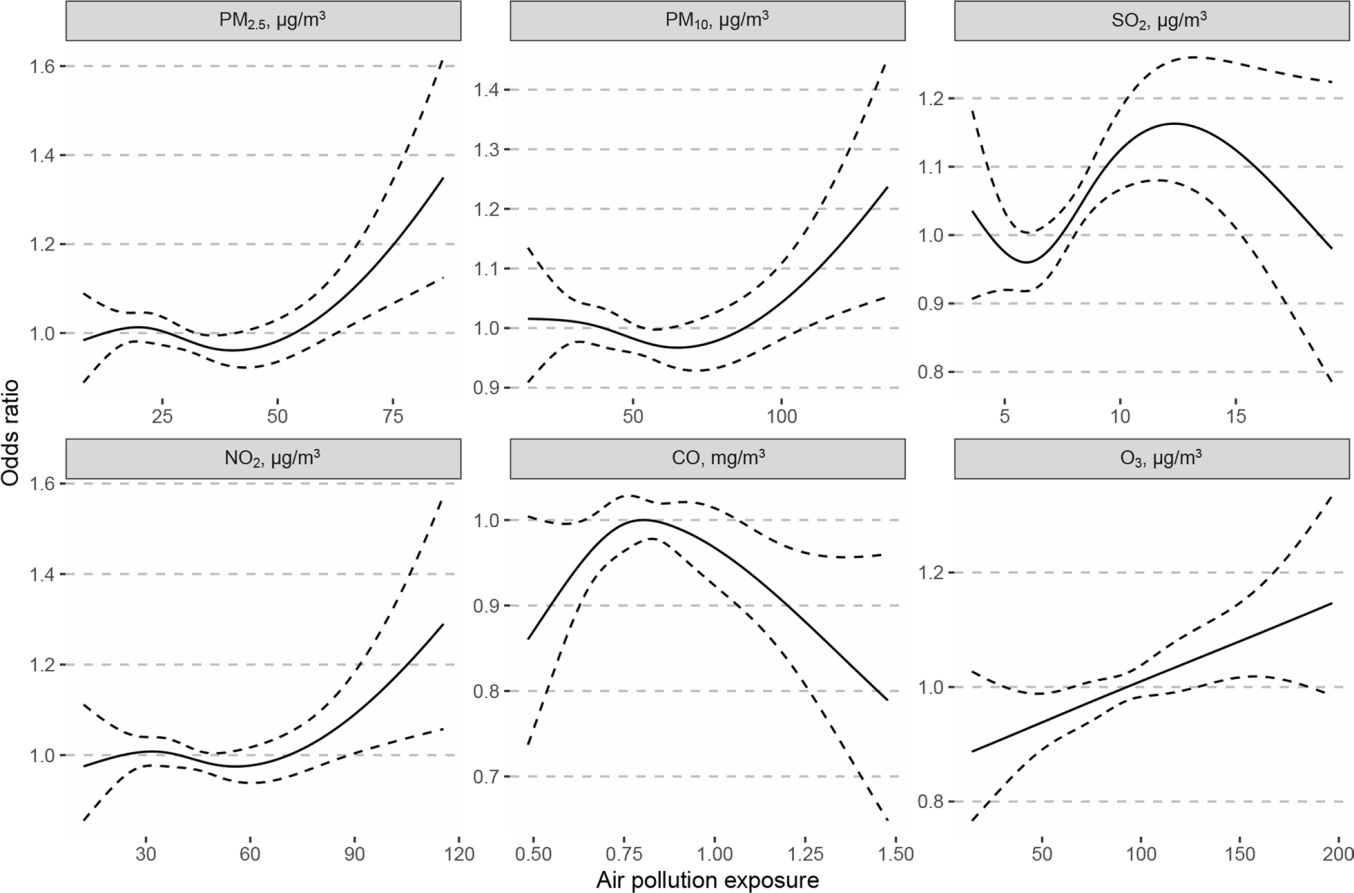
Exposure–response curves between air pollutant exposures and pneumonia hospital admission among COPD patients. Adjusted ORs (95% CIs) for PM_2.5_ (lag 2), PM_10_ (lag 2), SO_2_ (lag 03), NO_2_ (lag 03), CO (lag 2), and O_3_ (lag 04) were estimated for continuous exposures excluding those less and greater than 1% by using conditional logistic regression models, adjusting for temperature, relative humidity, and number of hospital admission. *OR* odds ratio, *CI* confidence interval, *COPD* chronic obstructive pulmonary disease, *PM*_*2.5*_ particulate matter with an aerodynamic diameter ≤ 2.5 µm, *PM*_*10*_ particulate matter with an aerodynamic diameter ≤ 10 µm, *SO*_*2*_ sulfur dioxide, *NO*_*2*_ nitrogen dioxide, *CO* carbon monoxide, *O*_*3*_ ozone

In single-pollutant model, we found significantly positive associations between exposure to PM_2.5_ (lag 2), SO_2_ (lag 1 to lag 3, lag 02 to lag 04), NO_2_ (lag 2, lag 02 to lag 04), O_3_ (lag 3 to lag 4, lag 03 to lag 04) and pneumonia hospital admissions among COPD patients, where the strongest association occurred at the lag 04 for SO_2_ (Fig. 1). An IQR increase of PM_2.5_ (lag 2; IQR: 22.1 μg/m^3^), SO_2_ (lag 03; IQR: 4.2 μg/m^3^), NO_2_ (lag 03; IQR: 21.4 μg/m^3^), and O_3_ (lag 04; IQR: 57.9 μg/m^3^) for pneumonia hospital admission among COPD patients was associated with an odds ratio of 1.043 (95% CI: 1.004, 1.083), 1.081 (95% CI: 1.026, 1.140), 1.045 (95% CI: 1.005, 1.088), and 1.080 (95% CI: 1.018, 1.147), respectively. The exposure–response curves with natural cubic spline function are shown in Fig. 2 and indicates significant nonlinear relationships for PM_2.5_, PM_10_, and SO_2_ on pneumonia hospital admission among COPD patients (*p* for nonlinear trend < 0.05). The curves were first flat at low concentrations then rose at high concentrations.

In stratified analysis, we did not observe that sex, age at hospital admission, or season modify the associations between exposures to air pollutants and pneumonia hospital admission among patients with COPD (all *p* for effect modification > 0.05; Additional file 1: Table S1). In two-pollutant models, the associations of SO_2_ with pneumonia hospital admissions remained significant after adjustment for PM or other gaseous pollutants (Additional file 1: Table S2). The associations for PM_2.5_, NO_2_, and O_3_ stay robust with additional adjustment, except that SO_2_ was taken as an adjustment item.

## Discussion

In this large time-stratified case-crossover study, short-term exposures to PM_2.5_, SO_2_, NO_2_, and O_3_ were significantly associated with pneumonia hospital admission among patients with COPD. We found that the odds ratios for pneumonia hospital admission among COPD patients with an IQR increase in ambient exposure of PM_2.5_, SO_2_, NO_2_, and O_3_ were 1.043, 1.081, 1.045, and 1.080, respectively. Nonlinear relation for PM_2.5_, PM_10_, and SO_2_ was observed with a flatten curves at low concentrations and steep at high concentrations. The associations were not modified by sex, age at hospital admission, and season at hospital admission.

According to the Global Burden of Disease Study, a great number of people suffered from COPD worldwide and COPD has been recognized as a fatal disease that caused about 0.9 million deaths in China in 2017 [2, 26]. COPD patients were found to have higher acute respiratory and systemic inflammation, and poorer lung function due to short-term exposure to air pollution, which indicated a plausible way for air pollution to trigger COPD exacerbations [27, 28]. Complications frequently accompanying COPD patients are thought to be responsible for poor life quality and survival [29]. As a common complication, the prognosis for pneumonia among patients with COPD were worse than those among the general population and the costs for health care due to pneumonia also placed heavy burdens on patients with COPD [30, 31]. We explored the acute adverse effects of ambient air pollution on pneumonia hospital admission among COPD patients. Our study raises novel sight in preventing COPD patients from admission for pneumonia by reducing levels of air pollution, and provides new evidence in terms of better health care management.

Ambient air pollution has been proved to be associated with hospital admission for pneumonia [13, 32]. Regarding PM_2.5_ and O_3_, Tian and colleagues conducted two time-series studies in China to assess the associations for particulate matter (PM) and O_3_ on pneumonia hospital admissions and found each 10 μg/m^3^ increase in PM_2.5_ and O_3_ were associated with 0.31% and 0.14% in percentage increase for pneumonia hospital admission, respectively [33, 34]. In another case-crossover study conducted in Shijiazhuang, China, Duan and colleagues observed that pneumonia hospital admission was positively correlated with exposure to PM_2.5_ (percent change: 1.1% per 10 μg/m^3^ increase) [35]. The results of our study are consistent with current studies. In addition, we noticed that no matter the ambient exposures of air pollutants in previous two studies were higher or lower than those in ours, the estimates of the associations in our study were much larger (percent changes for PM_2.5_ and O_3_: 1.9% and 1.3% per 10 μg/m^3^ increase, respectively). Harmful substances attached to PM and photochemical smog consisting of O_3_ were linked with pneumonia development [36]. Thus, given the airway characteristics of COPD patients, we speculated that COPD patients were more sensitive to ambient air pollution exposure than the general population.

Gaseous pollutants mainly affect respiratory health for COPD patients [37]. Apart from O_3_, a strong and stable association between exposure to SO_2_ and pneumonia hospital admission among COPD patients was observed from the two-pollutant models in our study (adjusted ORs ranging from 1.068 to 1.097). Moreover, we observed a significant difference between single-pollutant model of PM_2.5_, NO_2_, O_3_ and their two-pollutant models with adjustment for SO_2_, which indicated that their adverse effects may be attenuated by the coexistence of SO_2_. We therefore considered that SO_2_ may played an independent part in actual condition. However, Yee and colleagues conducted a systematic review and meta-analysis for acute effects of air pollution on pneumonia hospital admission and identified insignificant associations for SO_2_ in contrast to our results [38]. Significantly positive association for NO_2_ was also found. From our perspective, COPD patients were at relatively high risk for pneumonia hospital admission after exposures to gaseous pollutants. Airway epithelium serves as physical barrier to defend against air pollutants and airway epithelial dysfunction plays an important role in COPD initiation and progression [39]. Moist conditions in airway can better absorb gaseous pollutants and cause further irritation to epithelium. Defective epithelial barrier among COPD patients, including reduction in mucociliary clearance, macrophages, and natural killer cells, may lead to organisms more susceptible to infections such as bacteria and virus [36]. Collectively, coupled with previous studies, our study also speculated that short-term exposure to air pollution was associated with higher risk for pneumonia hospital admissions among patients with COPD.

Regarding PM_10_, previous evidence explored a positively significant association for pneumonia hospital admission [33, 40]. PM exposure was associated with an increase of inflammation-related cytokines, including interleukin-6, interleukin -12, interferon-γ, C-reactive protein and tumor necrosis factor-α [41]. The immunity alteration and oxidative stress induced by PM exposure may be responsible for the increased risk for pneumonia by impairment on macrophage function and mucociliary system [42, 43]. However, we did not observe significant association for PM_10_ on pneumonia hospital admission among COPD patients in the study. First, it was possibly due to the elderly population in the study. The elderly patients have experienced heavy air pollution in China in the past decades, which may account for the reduction of population susceptibility. In addition, the Chinese government released a series of air quality control policies in recent years and the ambient concentrations of various pollutants, especially PM_2.5_ and PM_10_, sharply dropped. In our results, we also only observed significant association for PM_2.5_ at lag 2 period and the lower limit of 95% confidence interval (1.004) was close to 1. The sustainable countermeasures may attenuate the effects for PM on pneumonia hospital admission. The flat at low concentrations and the steep shapes at high concentration in the exposure–response curve of air pollutants also supported our hypotheses.

One major strength of the present study was that we included a considerably large number of pneumonia admission cases to first investigate the association between exposure to air pollution and hospital admission for respiratory complication among patients with COPD. We collected the admission data from Guangdong province, the most populous province in China, during 2016–2019 in order to provide sufficient statistical power for further analyses. Second, compared to time-series study, the time-stratified case-crossover design performed in this study was convenient to control for potential confounding factors including subject-specific covariates (e.g., sex, age, smoking status) and time-variant variables (e.g., seasonality, long-term trend) by study design instead of modelling. Third, we applied the IDW method for individual-level exposure assessment on air pollution, which allowed us to assign the data from monitoring stations for all individuals based on personal residential addresses and to limit exposure misclassification. Finally, we carried out the main analyses among six ambient criteria air pollutants in China and performed two-pollutant models as sensitivity analysis in our study to assess the robustness of the associations between each air pollutant and pneumonia hospital admission among COPD patients.

There were also several limitations to acknowledge in our study. First, although the time-stratified case-crossover design was purported to control for potential confounding factors, some potential unmeasured confounding factors that changed over time remained uncertain effects on the associations. Second, daily data on air pollution were obtained from monitoring stations and individual-level exposures to air pollution were estimated using IDW method, which was designed to predict more accurate exposure estimates. However, the actual pattern of personal exposure to air pollution can be affected by a number of factors, including individual behavior in daily life and indoor air pollution exposure, where exposure misclassification may still be induced. Third, similar to some previous studies taking hospitalization as main outcome, the information on date of hospital admission was the unique factor for us to assign the case day [44]. Given that pneumonia may take time to develop from onset to admission after trigger exposures to air pollution, the date of hospital admission that did not stand for the true case day may still cause exposure misclassification. Finally, the population data consist of individuals aged ≥ 60 years from Guangdong province were included in our study, which limited the generalization of our results to other populations. However, the elderly accounted for the vast majority of COPD patients and the population in this study included all the COPD cases in Guangdong province.

## Conclusions

We found significant associations for short-term exposure to PM_2.5_, SO_2_, NO_2_, and O_3_ on pneumonia hospital admission among patients with COPD. It provides new evidence for comprehensive pneumonia prevention and treatment among COPD patients. Our findings also support several speculations and further studies are warranted for this field in the future to confirm our speculations.

## Supplementary Information

**Additional file 1: Table S1.** Adjusted ORs (95% CIs) for pneumonia hospital admission among patients with COPD associated with each IQR increase of exposure to PM_2.5_ (lag 2), PM_10_ (lag 2), SO_2_ (lag 03), NO_2_ (lag 03), CO (lag 2), and O_3_ (lag 04) stratified by sex, age, and season. **Table S2**. Adjusted ORs (95% CIs) for pneumonia hospital admission among patients with COPD associated with each IQR increase of exposure to PM_2.5_ (lag 2), PM_10_ (lag 2), SO_2_ (lag 03), NO_2_ (lag 03), CO (lag 2), and O_3_ (lag 04) estimated by single- and two-pollutant models. 

## Data Availability

The hospitalization data that support the findings of this study are available from Health Technology Identification and Human Resources Assessment Center in Guangdong, China but restrictions apply to the availability of these data, which were used under license for the current study, and so are not publicly available. The air pollution data and meteorological condition data used in this study can be downloaded from the website of National Urban Air Quality Real-Time Publishing Platform and the National Meteorological Information Center in China. The codes for analysis are available and can be requested from the corresponding authors.

## Abbreviations

CI: Confidence interval
CO: Carbon monoxide
COPD: Chronic obstructive pulmonary disease
df: Degree of freedom
ICD-10: International classification of diseases-10
IDW: Inverse distance weighting
IQR: Interquartile range
NO_2_: Nitrogen dioxide
O_3_: Ozone
OR: Odds ratio
PM: Particulate matter
PM_2.5_: Particulate matter with an aerodynamic diameter ≤ 2.5 μm
PM_10_: Particulate matter with an aerodynamic diameter ≤ 10 μm
SD: Standard deviation
SO_2_: Sulfur dioxide
