# Supplementary material for: Short-term exposure to ambient air pollution and pneumonia hospital admission among patients with COPD: a time-stratified case-crossover study
Source: Respir Res. 2022 Mar 26;23:71. doi: 10.1186/s12931-022-01989-9 (PMC8962484; doi:10.1186/s12931-022-01989-9)
Supplement: Supplementary file 1 — Additional file 1: Table S1. Adjusted ORs (95% CIs) for pneumonia hospital admission among patients with COPD associated with each IQR increase of exposure to PM2.5 (lag 2), PM10 (lag 2), SO2 (lag 03), NO2 (lag 03), CO (lag 2), and O3 (lag 04) stratified by sex, age, and season. Table S2. Adjusted ORs (95% CIs) for pneumonia hospital admission among patients with COPD associated with each IQR increase of exposure to PM2.5 (lag 2), PM10 (lag 2), SO2 (lag 03), NO2 (lag 03), CO (lag 2), and O3 (lag 04) estimated by single- and two-pollutant models. [file 12931_2022_1989_MOESM1_ESM.docx]

**Short-term exposure to ambient air pollution and pneumonia hospital admission among patients with COPD: a time-stratified case-crossover study**

Wenfeng Lu^1, 2, †^, Qi Tian^3, †^, Ruijun Xu^4^, Chenghui Zhong^2^, Lan Qiu^2^, Han Zhang^2^, Chunxiang Shi^5^, Yuewei Liu^4, *^, Yun Zhou^1, 2, *^

**Author Affiliations:**

^1^ *State Key Laboratory of Respiratory Disease, The First Affiliated Hospital of Guangzhou Medical University, Guangzhou, Guangdong 510120, China*

^2^ *School of Public Health, Guangzhou Medical University, Guangzhou, Guangdong 511436, China*

^3^ *Guangzhou Health Technology Identification & Human Resources Assessment Center, Guangzhou, Guangdong 510080, China*

^4^ *Department of Epidemiology, School of Public Health, Sun Yat-sen University, Guangzhou, Guangdong 510080, China*

^5^ *National Meteorological Information Center, China Meteorological Administration, Beijing 100081, China*

^*^ Correspondence: [liuyuewei@mail.sysu.edu.cn](mailto:liuyuewei@mail.sysu.edu.cn); [sarayunzhou@gmail.com](mailto:sarayunzhou@gmail.com)

^†^ These authors contributed equally to this work.

**Table S1** Adjusted ORs (95% CIs) for pneumonia hospital admission among patients with COPD associated with each IQR increase of exposure to PM_2.5_ (lag 2), PM_10_ (lag 2), SO_2_ (lag 03), NO_2_ (lag 03), CO (lag 2), and O_3_ (lag 04) stratified by sex, age, and season ^a^

| **Variable** | **PM_2.5_** | **PM_10_** | **SO_2_** | **NO_2_** | **CO** | **O_3_** |
| --- | --- | --- | --- | --- | --- | --- |
| Sex |  |  |  |  |  |  |
| Male | 1.043 (0.997, 1.092) | 1.036 (0.988, 1.086) | 1.095 (1.027, 1.167) | 1.058 (1.008, 1.110) | 1.020 (0.977, 1.064) | 1.055 (0.982, 1.134) |
| Female | 1.043 (0.975, 1.116) | 1.039 (0.969, 1.115) | 1.053 (0.960, 1.156) | 1.020 (0.952, 1.094) | 0.992 (0.929, 1.059) | 1.139 (1.022, 1.268) |
| *p* value ^c^ | 0.99 | 0.94 | 0.51 | 0.41 | 0.49 | 0.25 |
| Age |  |  |  |  |  |  |
| <85 yr | 1.032 (0.986, 1.079) | 1.028 (0.981, 1.077) | 1.082 (1.015, 1.153) | 1.040 (0.992, 1.091) | 1.001 (0.959, 1.045) | 1.089 (1.014, 1.169) |
| ≥85 yr | 1.071 (0.998, 1.149) | 1.060 (0.984, 1.141) | 1.089 (0.990, 1.197) | 1.060 (0.987, 1.139) | 1.037 (0.971, 1.107) | 1.060 (0.948, 1.184) |
| *p* value ^c^ | 0.38 | 0.50 | 0.91 | 0.67 | 0.38 | 0.69 |
| Season at hospital admission ^b^ |  |  |  |  |  |  |
| Cool | 1.068 (1.018, 1.121) | 1.071 (1.013, 1.132) | 1.138 (1.056, 1.227) | 1.079 (1.025, 1.135) | 1.010 (0.963, 1.059) | 1.144 (1.044, 1.254) |
| Warm | 1.025 (0.969, 1.086) | 1.017 (0.963, 1.074) | 1.068 (0.982, 1.161) | 1.018 (0.959, 1.081) | 1.022 (0.969, 1.077) | 1.073 (0.983, 1.170) |
| *p* value ^c^ | 0.29 | 0.19 | 0.27 | 0.15 | 0.75 | 0.32 |

Abbreviations: OR, odds ratio; CI, confidence interval; COPD, chronic obstructive pulmonary disease; PM_2.5_, particulate matter with an aerodynamic diameter ≤ 2.5 µm; PM_10_, particulate matter with an aerodynamic diameter ≤ 10 µm; SO_2_, sulfur dioxide; NO_2_, nitrogen dioxide; CO, carbon monoxide; O_3_, ozone.

^a^ ORs (95% CIs) were estimated using conditional logistic regression models, adjusting for temperature, relative humidity, and number of hospital admission. The IQR of PM_2.5_, PM_10_, SO_2_, NO_2_, CO, and O_3_ was 22.1 μg/m^3^, 34.6 μg/m^3^, 4.2 μg/m^3^, 21.4 μg/m^3^, 0.27 mg/m^3^, 57.9 μg/m^3^.

^b^ Warm season was defined as from May to October; Cool season: from November to April of next year.

^c^ *p* values were estimated by two-sample test and *p* value < 0.05 indicates significant effect modification.

**Table S2.** Adjusted ORs (95% CIs) for pneumonia hospital admission among patients with COPD associated with each IQR increase of exposure to PM_2.5_ (lag 2), PM_10_ (lag 2), SO_2_ (lag 03), NO_2_ (lag 03), CO (lag 2), and O_3_ (lag 04) estimated by single- and two-pollutant models ^a^

| Air pollutant | Model | OR (95% CI) | *p* value |
| --- | --- | --- | --- |
| PM_2.5_ | PM_2.5_ | 1.043 (1.004, 1.083) |  |
|  | PM_2.5_ + SO_2_ | 1.011 (0.965, 1.060) | 0.03 |
|  | PM_2.5_ + NO_2_ | 1.017 (0.964, 1.074) | 0.22 |
|  | PM_2.5_ + CO | 1.056 (1.007, 1.107) | 0.38 |
|  | PM_2.5_ + O_3_ | 1.034 (0.993, 1.077) | 0.30 |
| PM_10_ | PM_10_ | 1.037 (0.997, 1.079) |  |
|  | PM_10_ + SO_2_ | 1.000 (0.952, 1.050) | 0.02 |
|  | PM_10_ + NO_2_ | 0.995 (0.934, 1.061) | 0.11 |
|  | PM_10_ + CO | 1.047 (0.996, 1.101) | 0.53 |
|  | PM_10_ + O_3_ | 1.027 (0.985, 1.072) | 0.23 |
| SO_2_ | SO_2_ | 1.081 (1.026, 1.140) |  |
|  | SO_2_ + PM_2.5_ | 1.085 (1.018, 1.157) | 0.84 |
|  | SO_2_ + PM_10_ | 1.091 (1.022, 1.164) | 0.64 |
|  | SO_2_ + NO_2_ | 1.068 (1.002, 1.139) | 0.54 |
|  | SO_2_ + CO | 1.097 (1.037, 1.162) | 0.17 |
|  | SO_2_ + O_3_ | 1.072 (1.016, 1.132) | 0.19 |
| NO_2_ | NO_2_ | 1.045 (1.005, 1.088) |  |
|  | NO_2_ + PM_2.5_ | 1.049 (0.989, 1.113) | 0.85 |
|  | NO_2_ + PM_10_ | 1.066 (0.997, 1.139) | 0.47 |
|  | NO_2_ + SO_2_ | 1.015 (0.968, 1.066) | 0.04 |
|  | NO_2_ + CO | 1.068 (1.019, 1.120) | 0.10 |
|  | NO_2_ + O_3_ | 1.036 (0.994, 1.080) | 0.17 |
| CO | CO | 1.011 (0.976, 1.048) |  |
|  | CO + PM_2.5_ | 0.980 (0.937, 1.025) | 0.03 |
|  | CO + PM_10_ | 0.986 (0.942, 1.031) | 0.07 |
|  | CO + SO_2_ | 0.989 (0.951, 1.028) | 0.003 |
|  | CO + NO_2_ | 0.980 (0.939, 1.024) | 0.01 |
|  | CO + O_3_ | 1.008 (0.972, 1.045) | 0.09 |
| O_3_ | O_3_ | 1.080 (1.018, 1.147) |  |
|  | O_3_ + PM_2.5_ | 1.072 (1.005, 1.142) | 0.59 |
|  | O_3_ + PM_10_ | 1.074 (1.008, 1.144) | 0.71 |
|  | O_3_ + SO_2_ | 1.062 (0.999, 1.129) | 0.02 |
|  | O_3_ + NO_2_ | 1.066 (1.002, 1.134) | 0.17 |
|  | O_3_ + CO | 1.080 (1.017, 1.146) | 0.43 |

Abbreviations: OR, odds ratio; CI, confidence interval; COPD, chronic obstructive pulmonary disease; PM_2.5_, particulate matter with an aerodynamic diameter ≤ 2.5 μm; PM_10_, particulate matter with an aerodynamic diameter ≤ 10 μm; SO_2_, sulfur dioxide; NO_2_, nitrogen dioxide; CO, carbon monoxide; O_3_, ozone.

^a^ ORs (95% CIs) were estimated using conditional logistic regression models, adjusting for temperature, relative humidity, and number of hospital admission. The IQR of PM_2.5_, PM_10_, SO_2_, NO_2_, CO, and O_3_ was 22.1 μg/m^3^, 34.6 μg/m^3^, 4.2 μg/m^3^, 21.4 μg/m^3^, 0.27 mg/m^3^, 57.9 μg/m^3^.

^b^ *p* value was estimated by likelihood ratio test comparing the single- and nested two-pollutant model and *p* value < 0.05 indicates significance.
